# A Case Report on Advanced Squamous Cell Carcinoma of the Penis

**DOI:** 10.7759/cureus.30265

**Published:** 2022-10-13

**Authors:** Huy Q Nong, Andrew Sun, Niels-Jorgen Dyrved

**Affiliations:** 1 Internal Medicine, Methodist Dallas Medical Center, Dallas, USA; 2 Urology, Methodist Dallas Medical Center, Dallas, USA; 3 Pathology, Methodist Dallas Medical Center, Dallas, USA

**Keywords:** carcinoma of the penis, cancer, urology and oncology, urology, squamous cell carcinoma

## Abstract

This is a case of a 60-year-old Hispanic male with a history of poorly controlled diabetes who presented to the hospital with a chief complaint of a mass in the penis with mucopurulent discharge and drainage. The patient reported that the mass has been present for one year and had increased in size over the past six months. The patient had the mass biopsied at an outside surgical center one year ago, which was supposedly negative for cancer. On the initial physical examination, there was a large exophytic necrotic mass entirely replacing the penis with complete obliteration of the normal architecture of the glans and phallus with foul, purulent discharge. Significant bilateral palpable inguinal lymphadenopathy was present. A bedside biopsy was performed, which revealed squamous cell carcinoma (SCC). Computed tomography (CT) of the chest, abdomen, and pelvis was ordered for staging and revealed extensive pulmonary and hepatic metastasis, as well as bulky inguinal and retroperitoneal lymph node involvement. Systemic chemotherapy was offered to the patient; however, the patient declined and opted for hospice.

## Introduction

Penile cancer is a rare diagnosis in the United States with an incidence of less than one case per 100,000 males [1]. It is more common in developing countries such as parts of South America, Africa, and Asia. The majority of penile cancer is squamous cell carcinoma (SCC), which makes up 95% of cases. Squamous cell carcinoma is most often associated with human papillomavirus (HPV). Vaccination with HPV vaccines in adolescents has contributed to a low incidence of penile cancer in the United States [1].

The pathogenesis of penile cancer can be subdivided into HPV-dependent and HPV-independent pathways [2,3]. Penile SCC has been associated with high-risk HPV infections, particularly strains 16 and 18. HPV produces viral oncogenes E6 and E7, which target tumor suppressor genes and in turn lead to the malignant potential of infected squamous cells. These oncogenes are involved in the disruption of centrosome synthesis required for mitosis, resulting in premalignant and malignant HPV lesions [3].

HPV-independent penile cancers are commonly associated with a premalignant precursor lesion related to chronic inflammation [3]. The first sign of penile cancer is usually a localized penile lesion on the foreskin or glans [3]. Skin changes can include areas of thickening, ulceration, warty growth, or discharge and bleeding under the foreskin. Most cases of penile cancer are present in uncircumcised males. It is believed that poor hygiene with the buildup of smegma and chronic inflammation of the foreskin and glans contribute to the carcinogenesis of penile cancers. Male circumcision of the foreskin has been shown to be a protective factor in the development of penile cancer [3]. This case documents a rare presentation of penile SCC with advanced metastatic disease.

## Case presentation

History of presenting Illness

This is a case of a 60-year-old Hispanic male with a history of poorly controlled diabetes who presented to the hospital with a chief complaint of a mass in the penis. The patient reported that the mass has been present for one year and increased in size over the past six months. The penile mass was described as a painful discomfort. There was also a concern for urinary incontinence requiring adult diapers. The patient also complained of purulent mucous discharge coming from his penis. The patient denied any recent sexual contact over the past year and had not received an HPV vaccination.

Physical examination

**Figure 1:**
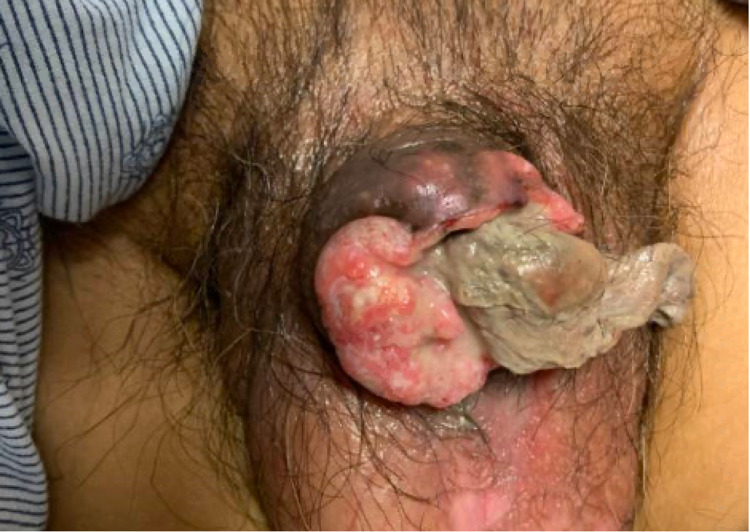
Advanced carcinoma of the penis presenting as an exophytic penile mass with necrosis and purulent discharge.

On physical examination, there was a large, exophytic, necrotic mass entirely replacing the penis with complete obliteration of the normal architecture of the glans and phallus with foul, purulent discharge (Figure 1). There was significant palpable inguinal lymphadenopathy bilaterally. The rest of his examination, however, was unremarkable.

Pathologic investigation

**Figure 2:**
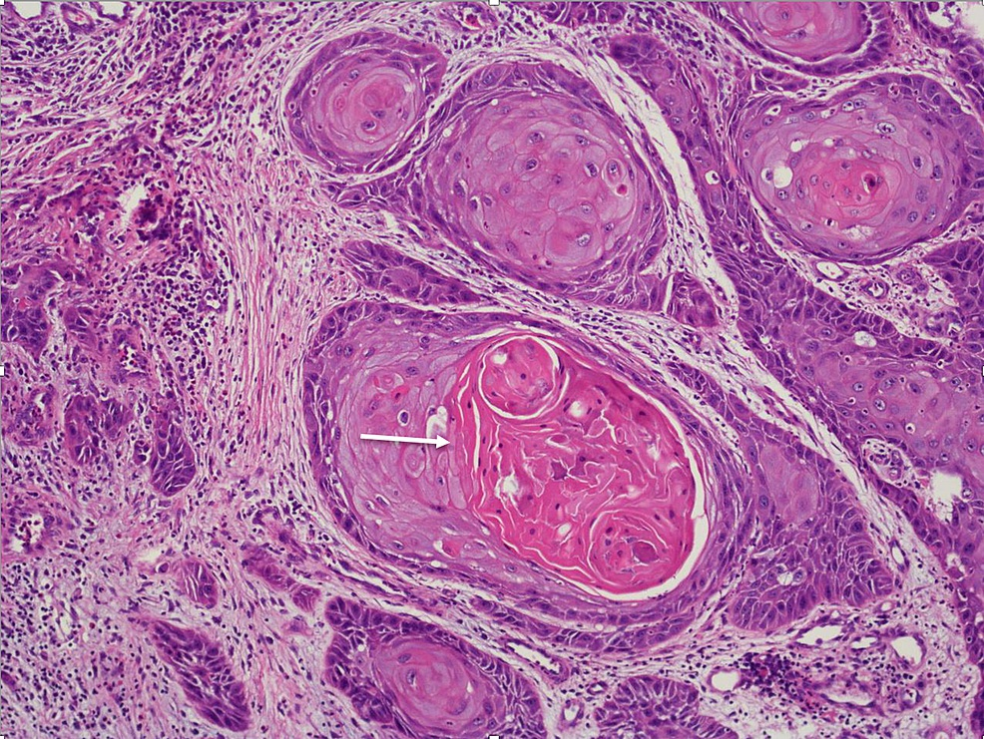
Malignant cells with brightly eosinophilic (i.e., pink) cytoplasm surround central areas of keratinization (arrow), a hallmark feature of squamous cell carcinoma.

**Figure 3:**
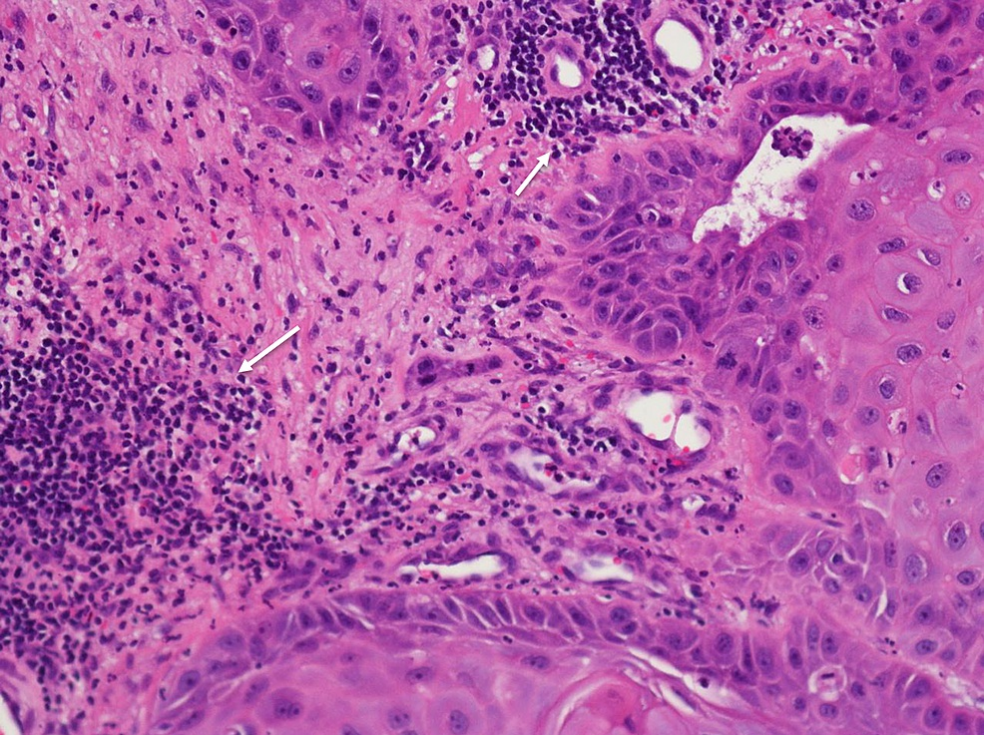
Desmoplastic response to a tumor with lymphocytic infiltration (arrows).

A bedside excisional biopsy was performed and sent for histopathologic examination, which revealed squamous cells with central areas of keratin pearls with brightly eosinophilic cytoplasm in invasive nests consistent with malignant squamous cell carcinoma (Figure 2). These malignant cells were well differentiated with keratin pearls and were extending to disoriented edges of the tissue sample, highly indicative of malignancy (Figure 3). Immunohistochemical (IHC) staining for p40 and p16 was negative.

Staging and treatment plan

**Figure 4:**
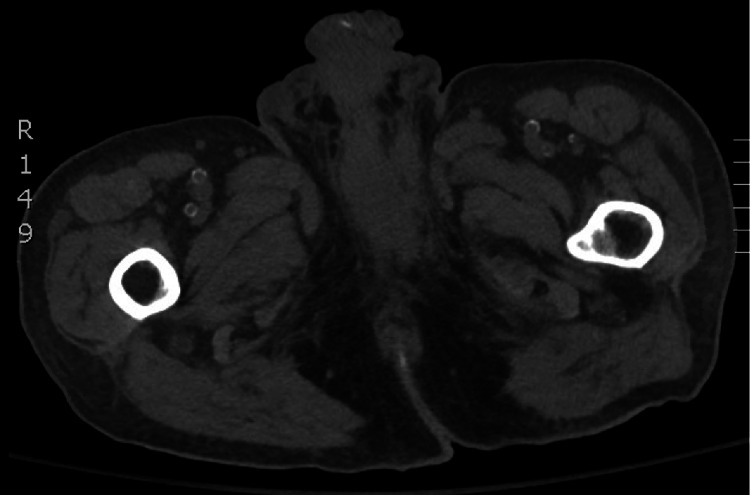
CT of the pelvis revealing marked abnormal appearance of the penis with obliteration of the normal penile architecture and an ulcerated, nodular, irregular appearance of the urethra. CT: computed tomography

**Figure 5:**
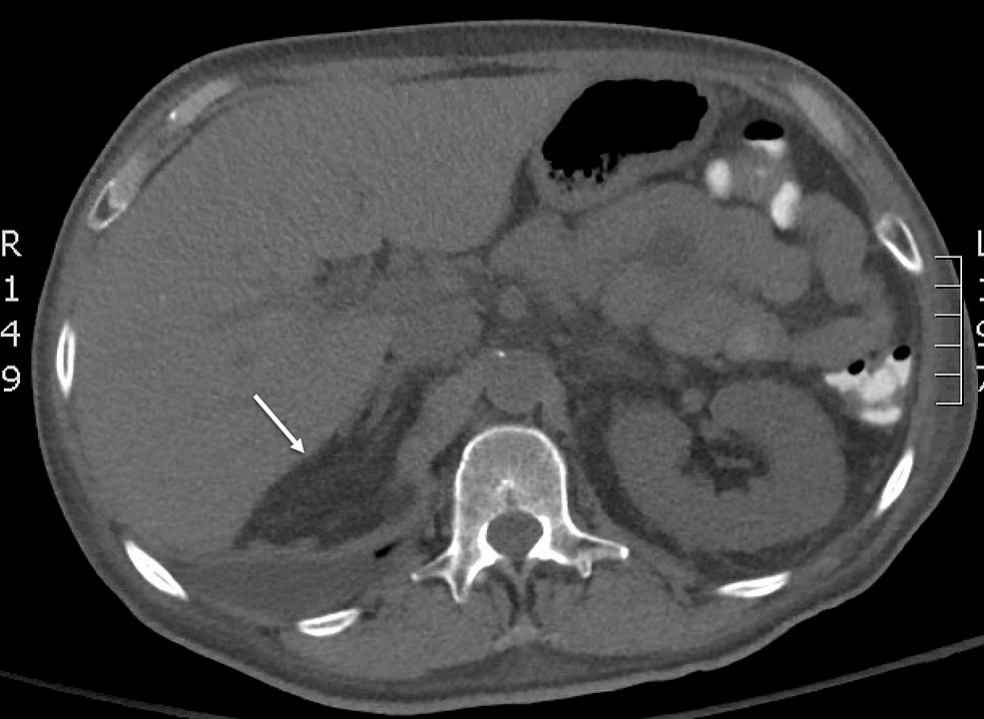
CT of the abdomen revealing malignant left hepatic lesion (arrow). CT: computed tomography

Computed tomography (CT) of the chest, abdomen, and pelvis was ordered for the staging of the patient’s cancer. The imaging revealed a large pelvic mass at the location of the phallus extending into the surrounding structures with disruption of the normal penile architecture. In addition, there was bulky inguinal and retroperitoneal lymph node involvement (Figure 4), along with extensive pulmonary and hepatic metastasis (Figure 5). Based on the investigative findings, the patient was staged as a stage IV, T4N3M1. Given the advanced stage of the cancer at the time of diagnosis, four rounds of systemic chemotherapy using a cisplatin-based regimen were planned for the patient as a means of palliative chemotherapy.

Expected and actual outcome

The expected outcome of this patient would have been very poor given the late nature of his disease. With systemic palliative chemotherapy, the patient would have expected to live with a median survival length of 12-18 months [2]. Without chemotherapy, the patient would have been expected to live for less than six months [3]. The patient had distant metastatic disease, unlikely to be responsive to chemotherapy [4]. The patient explored his options with chemotherapy and eventually decided to go home with hospice services.

## Discussion

When malignancy is suspected, abdominal and chest imaging is routinely ordered to evaluate for metastatic disease and staging. Neuroimaging, however, can be deferred unless neurologic symptoms are present, as penile malignancies seldom metastasize to the CNS. Lymph node dissection was deferred, as there was already evidence of metastatic disease. Treatment of the tumor is based on tumor size, location, and American Joint Committee on Cancer (AJCC) eighth edition tumor, node, and metastasis (TMN) stage [3].

The decision to treat SCC of the penis requires a multidisciplinary approach. While rare, the prognosis of metastatic penile carcinoma is poor. Despite appropriate treatment, the five-year survival is only 50% [1-3]. Unfortunately, delays in diagnosis of greater than six months are not uncommon. The main reasons include social stigma and the psychological consequence of describing the problem to their doctor or partner, insidious and nonspecific initial symptoms such as phimosis hiding the malignant lesion, and lack of awareness of the condition [3,5].

Penile carcinoma spreads via lymphatics, first to the inguinal lymph nodes and then to retroperitoneal and pelvic lymph nodes. Inguinal lymphadenopathy is a hallmark feature of penile carcinoma and is a factor in staging. Inguinal lymph nodes should be examined in any patient in whom penile cancer is suspected. Features of malignant lymphadenopathy include nodularity with hardening, well-circumscribed, or rubber-like texture of lymph nodes. Fixed inguinal lymphadenopathy is a poor prognostic factor [1].

For local tumors with no lymph node involvement, the recommendation is for penile-sparing surgeries such as simple excision, simple glansectomy, or Mohs micrographic surgery or local excision [1-3]. For patients with larger local tumors, partial or local penile amputation is recommended with negative margins due to the high risk of recurrence [4]. Patients with high-grade T1 disease and beyond often require more extensive surgical interventions such as partial or total penectomy [1,3,4].

Neoadjuvant chemotherapy (NAC) should be given for males with locally advanced unresectable penile carcinoma, bulky adenopathy, or evidence of pelvic nodal involvement with a goal of downstaging [1]. The main goal for NAC is to eliminate micrometastasis and shrink the tumor to facilitate surgery. The currently recommended regimen is four cycles of paclitaxel, ifosfamide, and cisplatin (TIP) [1].

For males with metastatic disease, treatment options are tailored based on functional status [6]. Outcomes for advanced disease remain poor with a median time of survival of 7-8 months [3]. Systemic chemotherapy is the standard of care for patients with unresectable locally advanced metastatic disease [3]. Enrollment in clinical trials should be considered. Given the poor prognosis, goals of care with an early discussion of palliative care are important [3]. Cisplatin-based regimens are most commonly used; however, no standard exists due to a lack of trials.

## Conclusions

Penile squamous cell carcinoma is a rare cancer and carries a poor prognosis overall. Given the patient’s demographics and origins from a Latin American country, a lack of previous HPV vaccination, and a personal history of uncircumcision, it is believed that his cancer is HPV-related; however, immunohistochemical staining was negative for HPV. It is important for clinicians to be aware of the subtle signs of cancerous growths of the penis, as early detection and recognition afford a better prognosis. Initial symptoms are often nonspecific such as verrucous growths, bleeding lesions under the glans, or phimosis hiding the malignant lesion. Management is multidisciplinary following pathologic confirmation and typically involves both surgery and chemotherapy depending on tumor size, location, lymph node involvement, and the patient’s performance status.

It is important to recognize the psychological impact of this disease, especially with penectomy and the resulting disfigurement of the normal male anatomy. Self-esteem issues, anxiety, depression, and lack of sexual satisfaction are all associated with penile carcinoma. These psychological associations should also be taken into consideration by the patient.
